# Research on Multiple-AUVs Collaborative Detection and Surrounding Attack Simulation

**DOI:** 10.3390/s24020437

**Published:** 2024-01-10

**Authors:** Zhiwen Wen, Zhong Wang, Daming Zhou, Dezhou Qin, Yichen Jiang, Junchang Liu, Huachao Dong

**Affiliations:** 1 Xi’an Precision Machinery Research Institute, Xi’an 710077, China; shanda623@163.com; 2 School of Astronautics, Northwestern Polytechnical University, Xi’an 710072, China; daming.zhou@nwpu.edu.cn; 3 School of Marine Science and Technology, Northwestern Polytechnical University, Xi’an 710072, China; qindezhou@mail.nwpu.edu.cn (D.Q.); jyc_writer@mail.nwpu.edu.cn (Y.J.); liujunchang_nwpu@mail.nwpu.edu.cn (J.L.); hdong@nwpu.edu.cn (H.D.)

**Keywords:** AUV formation, path planning, collaborative detection, collaborative surrounding attack, LSTM, artificial potential field

## Abstract

Due to limitations in operational scope and efficiency, a single Autonomous Underwater Vehicle (AUV) falls short of meeting the demands of the contemporary marine working environment. Consequently, there is a growing interest in the coordination of multiple AUVs. To address the requirements of coordinated missions, this paper proposes a comprehensive solution for the coordinated development of multi-AUV formations, encompassing long-range ferrying, coordinated detection, and surrounding attack. In the initial phase, detection devices are deactivated, employing a path planning method based on the Rapidly Exploring Random Tree (RRT) algorithm to ensure collision-free AUV movement. During the coordinated detection phase, an artificial potential field method is applied to maintain AUV formation integrity and avoid obstacles, dynamically updating environmental probability based on formation movement. In the coordinated surroundings attack stage, predictive capabilities are enhanced using Long Short-Term Memory (LSTM) networks and reinforcement learning. Specifically, LSTM forecasts the target’s position, while the Deep Deterministic Policy Gradient (DDPG) method controls AUV formation. The effectiveness of this coordinated solution is validated through an integrated simulation trajectory.

## 1. Introduction

The unmanned, intelligent, multi-functional, and adaptable traits of AUVs have garnered significant global attention, establishing them as crucial carrier platforms for executing underwater missions [1]. Given the intricacies of the underwater environment and the escalating mission requirements, relying on a single AUV becomes challenging. Consequently, collaborative task execution by multiple AUVs emerges as an inevitable trend [2]. Compared to individual AUVs, multiple AUVs demonstrate superior adaptability and responsiveness when facing complex underwater environments and advanced tasks [3]. The advantages of multiple AUV collaborative systems are evident, not only expanding perceptual range and enhancing efficiency but also finding applications in diverse areas such as ocean data collection, seabed exploration, and underwater target search [4]. Therefore, in underwater environments, integrating multiple AUV systems is indispensable for future AUV research [5]. 

Recent years have witnessed comprehensive developments in various technical aspects of multi-AUV formations, including research on path planning, formation control, and cooperative capture. For instance: Zhang et al. [6] proposed an AMPSO algorithm for the three-dimensional path planning of multiple AUVs, aiming to enhance the speed and exploration capability of autonomous path planners for multiple AUVs. Hu et al. [7] proposed a Formation Comprehensive Cost (FCC) model to achieve collision avoidance within a formation by balancing convergence time, transformation distance, and sensor network power consumption. Qin et al. [8] achieved collision-free and coordinated motion for each AUV by integrating master-slave formation control methods with a path planning strategy based on artificial potential fields. Wen et al. [9] proposed a novel controller, composed of an improved artificial potential field, graph rigidity theory, and affine transformation (IAPF-GR-AT), to achieve real-time formation reconstruction and obstacle avoidance for AUV formations in a three-dimensional marine environment. Huang et al. [10] proposed a distance-based negotiation approach to control AUV formations to perform target encirclement. Liang et al. [11] successfully addressed the challenge of coordinated encircling of targets by AUV formations with varying motion capabilities through the application of a heuristic neural network approach. Cao et al. [12] proposed a multi-AUV search algorithm based on dynamic prediction of the target’s motion trajectory. This allows AUV formation members to swiftly reach the desired hunting points, facilitating efficient pursuit and capture of the target. Petritoli et al. [13] demonstrated the behavioral simulation of a dispersed fleet of underwater unmanned submersibles in a confined maritime area. In summary, AUV collaborative formation research has a wide range of fields, such as path planning, formation control, and formation encirclement. 

However, existing research on AUV formation tasks tends to be fragmented, lacking a comprehensive process for the entire execution of AUV formation missions. This paper addresses this gap by presenting a complete process for simulating AUV formation tasks, divided into four stages: The first simulation stage is the long-range ferry phase, the AUV formation sails to the designated area according to the path planned by the RRT. The second simulation stage is the collaborative search phase. Once the formation reaches the suspicious target area, the AUV formation collaboratively searches for the target. The third simulation stage is the cooperative surrounding attack phase. In this stage, the AUV formation predicts the target trajectory using the LSTM method. The fourth simulation stage is the capture phase, the AUV formation employs the DDPG method to control individual AUVs for target capture. This comprehensive research framework has the potential to provide valuable guidance and insights into the integrated execution of AUV formation missions.

The innovations of this paper are as follows:

1. The entire process of AUV formation execution and integrating methods is developed for four different simulation stages of AUV formation;
2. The artificial potential field method is effectively employed to calculate obstacle avoidance waypoints for the AUV formation;
3. The utilization of LSTM neural networks efficiently predicts the motion trajectories of targets, and the DDPG method is introduced for AUV formation control and a high success rate of the surrounding attack can be achieved.

The remaining sections of this paper are as follows: Section 2 introduces the detailed methods that address the requirements of the entail task, including the AUV motion equations, path planning algorithm, dynamic obstacle avoidance method, target state trajectory prediction method, and collaborative rounding method. Then in Section 3, the entire collection of AUV forms is simulated. The conclusion is presented in Section 4.

## 2. Methodology

**Figure 1:**
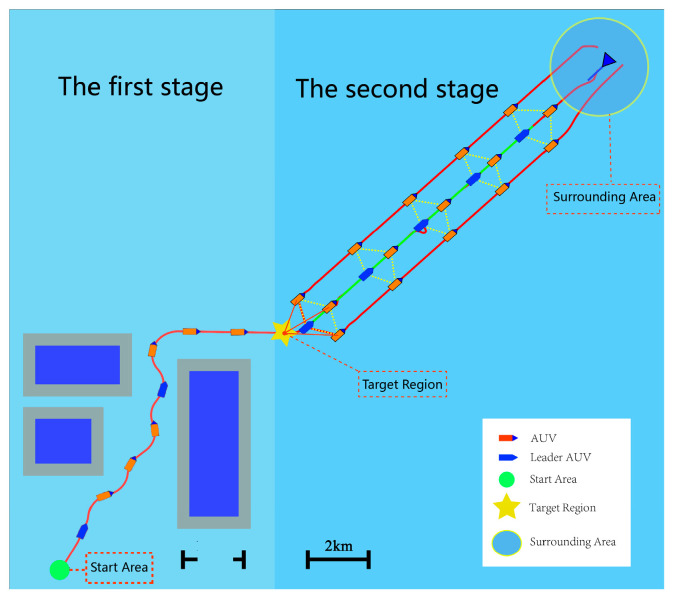
A complete simulation process of multi-AUVs formation execution mission. The simulation process delineates the complete execution of a multi-AUV formation mission. The diagram bifurcates into two segments. The left side portrays the AUV fleet engaging in a long-range transit, moving from the starting point towards the target point, preparing for the collaborative search phase. On the right side, subsequent to reaching the target point, the AUV fleet initiates a collaborative search employing the optimized formation. Upon target detection, the fleet predicts the target’s trajectory, facilitating interception.

Figure 1 illustrates the configuration of the multi-AUV formation, initially arranged linearly from the starting position. The formation utilizes the RRT algorithm to navigate around static obstacles, ultimately reaching the designated area. Subsequently, the formation transitions into a diamond configuration, employing the artificial potential field method to detect suspicious areas while accounting for communication limitations. Upon successful target detection, the LSTM algorithm facilitates dynamic trajectory prediction. The AUV formation initiates a search to identify optimal interception positions. Upon locating a defined position, the formation converges and engages the AUV to fulfill the assigned mission. The algorithm framework for the entire task is depicted in Algorithm 1.

**Figure 2:**
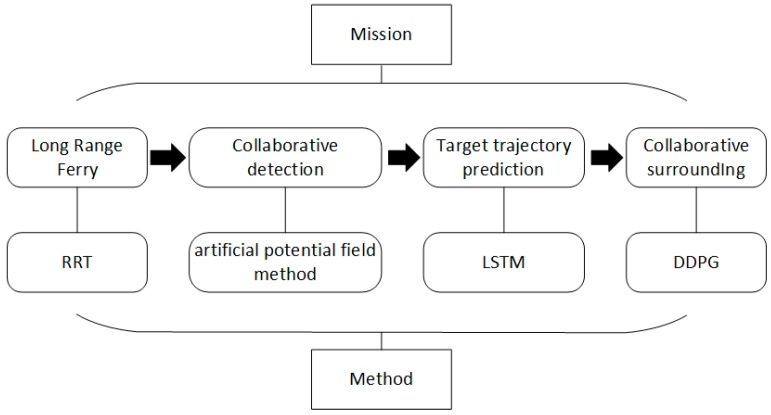
The proposed method consists of long-range ferry, collaborative detection, target trajectory prediction, and collaborative surrounding four stages.

To meet the task requirements outlined in Figure 1, this section, illustrated in Figure 2, breaks down the comprehensive mission requirements into four categories: Remote Ferries, Collaborative Detection, Target Trajectory Prediction, and Collaborative Environments. More precisely, we apply the RRT approach to fulfill the mission requirements for long-distance ferries. Artificial potential field methods are employed for collaborative detection. LSTM is utilized for predicting target trajectories, and DDPG methods are implemented to address collaboration-related requirements.
**Algorithm 1:** Multiple-AUVs Collaborative Detection and Surrounding attack Simulation/*Initialization*/ (01)
Env  ←  Construct virtual environment; /*First Stage*/(02) 
$S_{target}$  ←   Sample randomly (Env , 1);(03) 
$S_{cons}$  ←  Obstacle information;(04) For k = 1: *K*/*K is the number of the AUVs*/(05)  
$S_{k}$  ←   Sample randomly (Env , *k*); (06)  
$Ferry\;path_{k}$  ←  $\mathrm{RRT}\;(S_{target}$ $,\;S_{k}$ $,\;S_{cons}$ ); (07) End For /* Second Stage */ (08) /* Determine the topology of AUVs formation */ (09) 
$\theta_{model}$  ←  Build parametric formation model; (10) 
Obj  ←  Construct a multi-objective optimization problem (Env , $\theta_{model}$ ); (11) 
$\theta_{model}^{*}$  ←  $\;\mathrm{Optimize}\;\mathrm{based}\;\mathrm{on}\;\mathrm{PSO}\;\mathrm{and}\;\mathrm{GA}\;(\theta_{model}$ , Obj );(12) 
Search path  ←  $\;\mathrm{Generation}\;\mathrm{path}\;(\theta_{model}^{*}$ );
/* Third Stage */ (13) 
$Env_{surround}$  ←   Build training environment (Env $,\;S_{target}$ );(14) *D*  ←  $\;\mathrm{Construct}\;\mathrm{training}\;\mathrm{data}\;(Env_{surround}$ , *Batch size*); (15) 
LSTM model  ←  Training model (*D*); (16) For k = 1: *K*(17)    
$Net_{actor}^{k}$  ←  $\mathrm{DDPG}\;(S_{target}$ $,\;S_{k}$ , LSTM model ); (18) End For (19) 
Surround path  ←  $\;\mathrm{Co}-\mathrm{Simulate}\;(Net_{actor}$ );

### 2.1. AUV Motion Equations

Before establishing the entire task model, the AUV motion equation system is built according to the momentum theorem and the momentum moment theorem, as stated in the definition of reference [14].

(1) $$[\begin{array}{l} \dot{x}\\ \dot{y}\\ \dot{z} \end{array}]=C_{0}^{b} [\begin{array}{l} v_{x}\\ v_{y}\\ v_{z} \end{array}]$$

(2) $$[\begin{array}{l} {\dot{v}}_{x}\\ {\dot{v}}_{y}\\ {\dot{v}}_{z}\\ {\dot{\omega }}_{x}\\ {\dot{\omega }}_{y}\\ {\dot{\omega }}_{z} \end{array}]=A_{m}{\;\;}^{-1} [\begin{array}{l} f_{s}+f_{g}+f_{t}+f_{tl}\\ m_{s}+m_{g}+m_{t} \end{array}]$$

(3) $$[\begin{array}{l} \dot{\theta }\\ \dot{\varphi }\\ \dot{\psi } \end{array}]= [\begin{array}{l} 0\;\cos\varphi /\cos\theta \;-\sin\varphi /\cos\theta \\ 0\;\;\;\sin\varphi \;\;\;\cos\varphi \\ 1\;-\cos\varphi \tan\theta \;\sin\varphi \tan\theta \end{array}] [\begin{array}{l} w_{x}\\ w_{y}\\ w_{z} \end{array}]$$

The Equations (1)–(3) together form a set of AUV spatial kinematic equations comprising 12 equations. x y z represent the displacement of the AUV in three directions within the inertial coordinate system. $v_{x}$ $v_{y}$ $v_{z}$ signifies the AUV’s velocity in three directions within the body coordinate system. $w_{x}$ $w_{y}$ $w_{z}$ denotes the rotational angular velocities of the AUV around the x, y, and z axes in the body coordinate system. $C_{0}^{b}$ is the matrix for transforming the body coordinate system to the inertial coordinate system. $A_{m}$ denotes the added mass matrix. $f_{s}$ and $f_{g}$ signify the forces exerted by the fluid and the buoyant forces on the AUV, respectively. $f_{t}$ is the force transformed by mass-based coordinates. $f_{tl}$ indicates the propeller thrust. $m_{s}$ is the moment of hydrodynamic force acting on the AUV. $m_{g}$ represents the gravitational torque acting on the AUV. $m_{t}$ illustrates the moment (torque) transformed by mass-based coordinates. θ , ψ , and φ denote the tilt angle, the yaw angle, and the roll angle of the AUV. For specific information regarding coordinate system definitions and parameters, please refer to [14].

### 2.2. Path Planning

The underwater realm is characterized by intricate, and unevenly distributed complex environments. In addressing this, a practical and efficient motion planning approach is introduced based on RRT. This technique involves generating collision-free trees through random processes, enabling thorough exploration of the vicinity surrounding an AUV.

**Figure 3:**
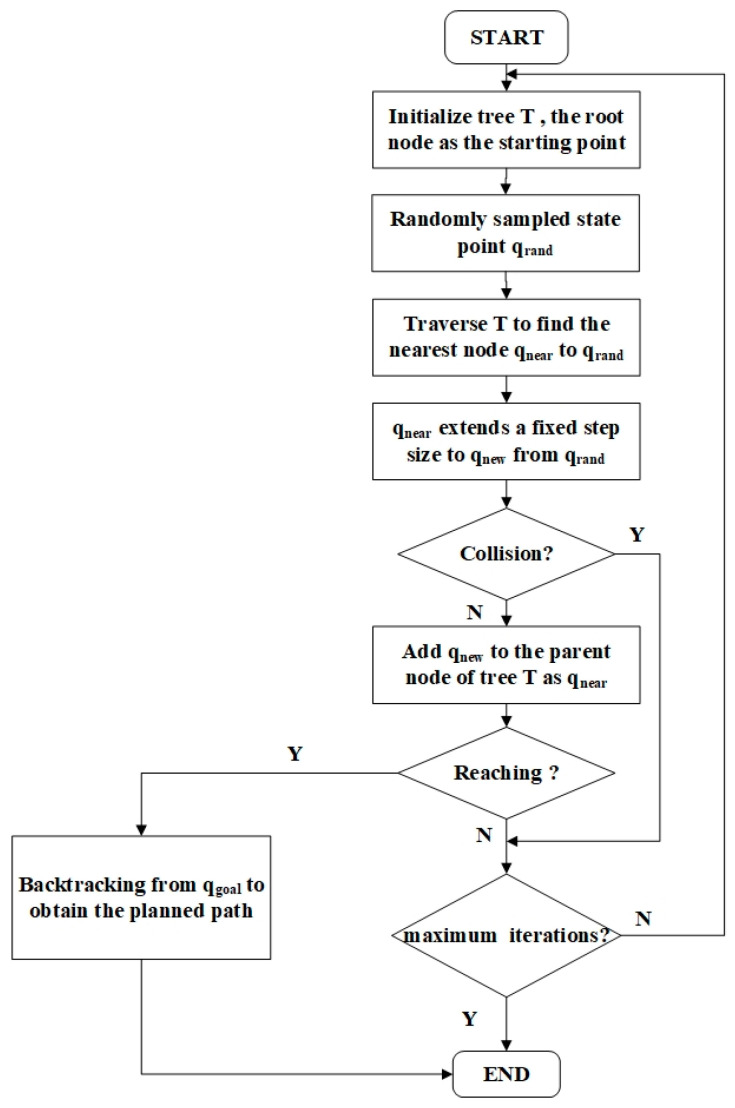
RRT flowchart. In the initial phase of the algorithm, RRT designates the starting point as the root node and then randomly samples a point $q_{rand}$ . Subsequently, RRT identifies the nearest node $q_{near}$ , in the tree structure and establishes a connection between $q_{near}$ and $q_{rand}$ . If the path between $q_{new}$ and $q_{rand}$ does not intersect with obstacles, RRT adds $q_{new}$ to the tree. During each iteration, RRT checks if the new node has reached the goal. If so, RRT generates the final path and concludes the algorithm. This iterative process involves continuously expanding new nodes in the tree to rapidly explore feasible paths until reaching the specified number of iterations or satisfying the termination conditions.

As depicted in Figure 3, the path-planning method that relies on sampling is commonly known as the RRT algorithm [15]. A tree originates from its root node and extends branches, eventually reaching endpoints. At this stage, a distinctive path can be traced back to the root node. The process begins by initializing the tree and defining the start and end points. Subsequently, a sampling function is executed, where the endpoints are selected as sampling points with a predetermined probability. This sample point explores the neighboring region to identify the nearest node, at which the tree is further expanded. A line is drawn to connect the sample point and its closest node. The existing branch is discarded if it intersects with an obstacle, and a fresh sampling is conducted to identify a new nearest node, thus ensuring obstacle avoidance. As the sampling process progresses, a new node is created, and all existing nodes including the branch nodes and the root node are considered together. This cumulative set of nodes guides the ongoing sampling. This process continues to advance and gradually approaches the endpoint. The distance from the node to the endpoint is calculated to determine whether the node can effectively reach the target point. Additionally, it evaluates whether the step length is reasonable and devoid of obstacles. Subsequently, a path is drawn between the node and the target point to ensure a connection.

**Figure 4:**
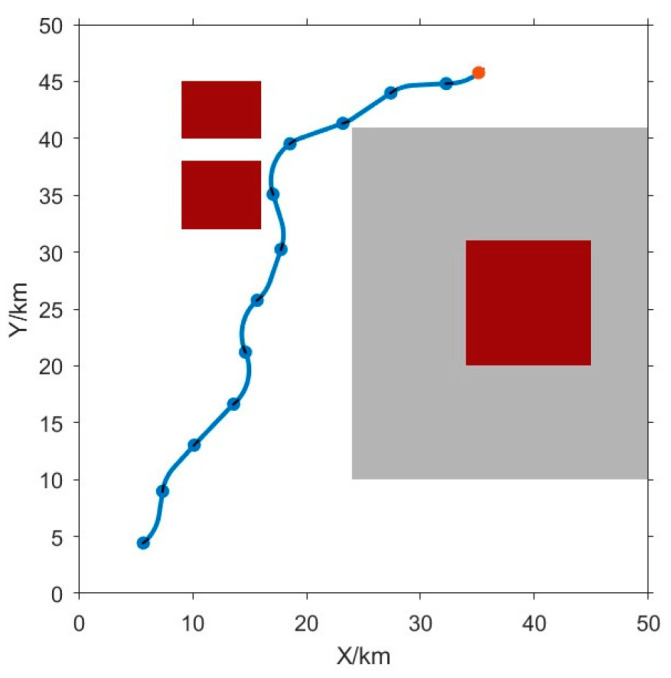
The RRT (Fast Random Tree Method) planning path. The red point signifies the target point, the blue point represents the AUV, and the blue curve out-lines the planned trajectory. The gray area denotes the obstacle zone with a weight of 0.5, while the red area signifies the obstacle area with a weight of 0.9.

As depicted in Figure 4, obstacles exhibit distinct starting and ending positions. The blue dot indicates the position of the vehicle, while the gray regions indicate obstacle zones with values between 0.5 and 0.9. The red area signifies obstacle locations with a weight exceeding 0.9. The blue curve delineates the path devised by the RRT algorithm.

### 2.3. Dynamic Obstacle Avoidance

During this mission phase, the task area is occupied by continuously moving noise source obstacles denoted as $O_{j}$ . These obstacles are strategically placed by the opponent to intentionally disrupt operations within the designated area. In alignment with the mission’s directives, the artificial potential field approach is used to address dynamic obstacle avoidance. We assign each obstacle a separate force field. Specifically, when an obstacle falls within the detection range of $A_{i}$ (the *i*th AUV), the associated potential field exerts a repulsive influence. The formula for calculating the force is as follows:

(4) $$F_{ij}=\alpha \left(\frac{1}{D_{ij}}-\frac{1}{D_{0}}\right)\frac{1}{D_{ij}^{2}}$$

where $D_{ij}$ means the distance between $A_{i}$ and $O_{j}$ , $D_{0}$ means the safety distance, and α is the repulsion coefficient.

The obstacle avoidance repulsion can be obtained by summing the repulsive forces of each obstacle $O_{j}$ , then the expected acceleration of $A_{i}$ can be calculated by:

(5) $$F_{i}=\sum_{j}F_{ij}=m\dot{v}$$

**Figure 5:**
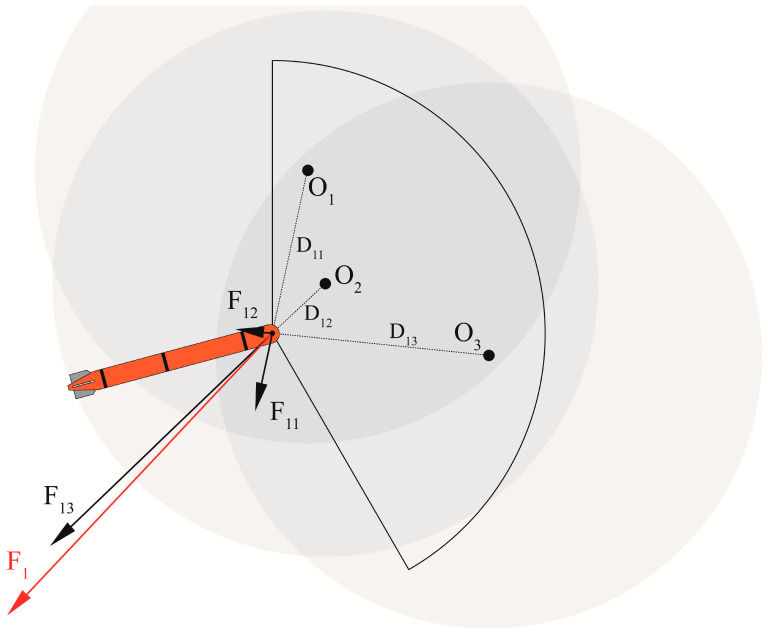
Artificial potential field obstacle avoidance principle. The three black dots symbolize the obstacles, with the fan-shaped area representing the AUV’s detection range. The three black arrows illustrate the repulsive forces exerted by the obstacles on the AUV, while the red arrow represents the combined force resulting from these three repulsive forces.

As depicted in Figure 5, three dynamic obstacles within the sector detection range are assigned individual potential fields. Varying forces are applied to the vehicle based on the distance from each obstacle. The cumulative force represents the vehicle’s obstacle avoidance repulsion force. By introducing the distance into Equation (4), the resultant force of the force field can be calculated on the AUV. Furthermore, to convert the resultant force into the control quantity, the expected acceleration of the AUV can be obtained by using Newton’s second law (Formula (5)).

### 2.4. Cooperative Detection

**Figure 6:**
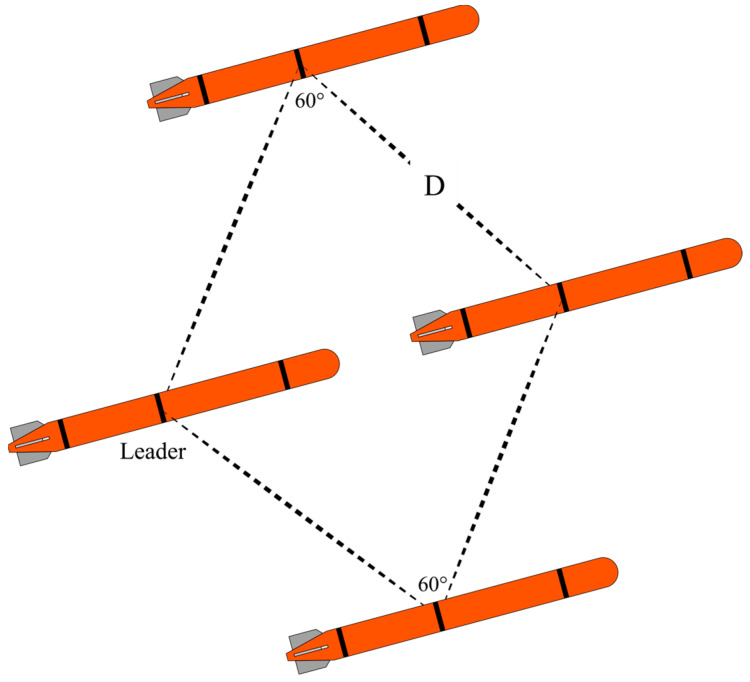
Formation structure. D represents the communication distance between AUVs.

The formation of AUVs can greatly enhance the efficiency and accuracy of search operations [16]. Therefore, to fulfill the mission objectives, a strategy is implemented for involving the formation of four AUVs. A transverse diamond formation with an acute angle of 60° is configured and the maximum communication distance is 2000 m. In this arrangement, the Leader AUV is positioned at the rear, as illustrated in Figure 6.

The environment is divided into multiple grids $C_{k}$ , each grid is assigned a probability value representing the probability that the formation judges the presence of the target [17]. This probability value is derived from the environment probability obtained by each formation member through exploration $P_{ik}$ . Followers transmit the data to the leader, and then the leader synthetically calculates the environmental probabilities $Q_{k}$ .

The Shannon entropy is employed as a metric to quantify the level of uncertainty within an area during the exploration process:

(6) $$\chi_{k}=-\frac{Q_{k}logQ_{k}+\left(1-Q_{k}\right)log\left(1-Q_{k}\right)}{log2}\;$$

**Figure 7:**
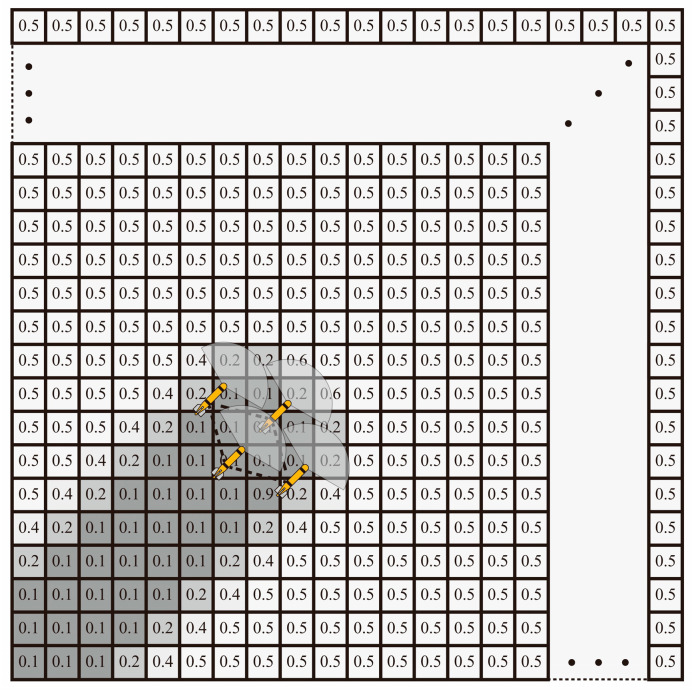
Environment probabilities. After the AUV formation traverses a specific area, the information entropy map is updated based on the detection information gathered by the AUV fleet. A probability of 1 or 0 indicates that the information about the target in that region is certain, signifying the presence or absence of a target, respectively. A probability of 0.5 signifies the highest level of uncertainty in the information for that area, representing a completely unknown state. The dashed areas signify more distant environmental regions.

As depicted in Figure 7, based on the given expression, probabilities tend to be categorized as “certain” when they are near 0 or 1. In such cases, the resulting Shannon entropy, which is calculated by Formula (6), is low. Conversely, probabilities close to 0.5 indicate the highest level of uncertainty, resulting in the highest Shannon entropy value.

**Figure 8:**
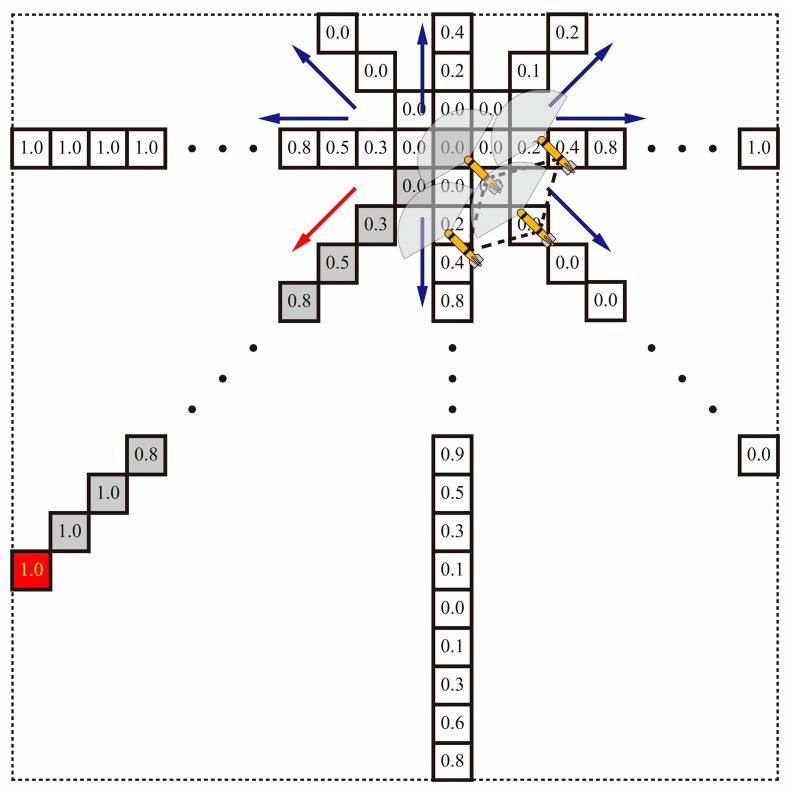
Target localization for cooperative detection tasks based on Shannon entropy. The red arrow indicates the direction in which the target is more likely to move, and the blue arrow indicates the direction in which the target is less likely to move.

As depicted in Figure 8, after a mission cycle, when the formation transitions into cruise mode, it employs a random selection from eight potential directions. The formation then moves toward the region that is furthest away and exhibits the highest Shannon entropy in the chosen direction. This criterion requires continuous updating of cruise routes, in order to obtain comprehensive global knowledge. 

The path originating from the lower right corner signifies the concluding work cycle. During this phase, a new direction is selected toward the bottom left once the formation has successfully reached the target area and completed the mission, indicated by the red arrow. Along this chosen path, the maximum entropy value is 1.0, and the location with the highest entropy value of 1 is selected as the target position for the subsequent task cycle, which is represented by the red grid.

### 2.5. Target State Prediction Method Based on LSTM

The Long Short Memory Network (LSTM) [18] primarily addresses the challenge of long-term dependencies encountered in conventional Recurrent Neural Networks (RNNs). This type of network is commonly employed for tasks such as classification and prediction [19].

The mission requirements outlined in this paper pose a significant challenge for the communication and detection of AUV formations. Recognizing LSTM’s generalization and processing capabilities in handling irregular data as an effective time sequences processing tool, we incorporate it as the target trajectory prediction method in the third stage within the task. What is more, the AUV formation cannot continuously detect the target trajectory under some working conditions, so LSTM is needed to predict the target position in order to detect the target trajectory.

**Figure 9:**
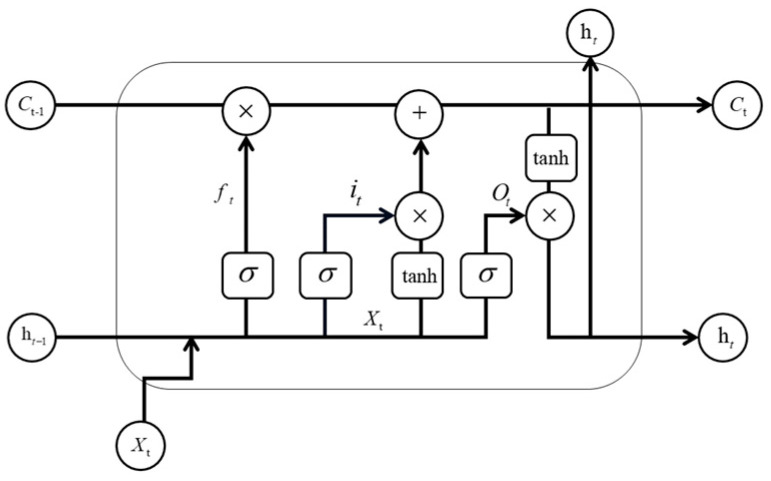
Single-layer LSTM structure diagram.

In Figure 9, the entire network comprises a forgetting gate $f_{t}$ , an input gate $i_{t}$ , and an output gate $o_{t}$ . By using Formula (7), the network takes the target’s current motion state as input, and then processes it to predict and determine the future motion state of the target. The calculation formula is as follows:

(7) $$\left\{\begin{array}{l} i_{t}=\sigma (W_{i}\cdot [h_{t-1},x_{t}]+b_{i})\\ f_{t}=\sigma (W_{f}\cdot [h_{t-1},x_{t}]+b_{f})\\ {\tilde{C}}_{t}=\tanh(W_{C}\;\cdot [h_{t-1},x_{t}]+b_{C})\\ o_{t}=\sigma (W_{o}\cdot [h_{t-1},x_{t}]+b_{o})\\ C_{t}=f_{t}*C_{t-1}+i_{t}*{\tilde{C}}_{t}\\ h_{t}=o_{t}*\tanh(C_{t}) \end{array}\right.$$

**Figure 10:**
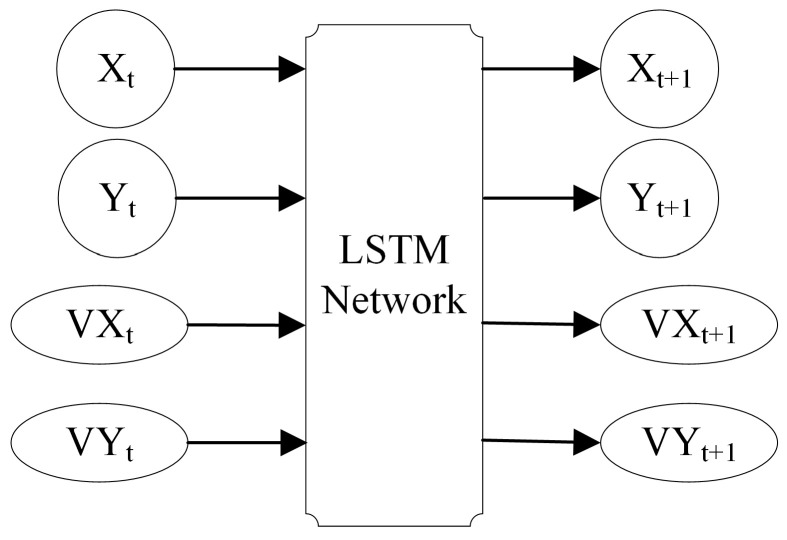
The input and the output of the LSTM network.

In Figure 10, a target exhibiting distinct motion characteristics possesses a connection between its current state and its future state. 

### 2.6. Collaborative Rounding Method Based on DDPG

#### 2.6.1. DDPG

DDPG (Deep Deterministic Policy Gradient) is introduced to tackle challenges in continuous action control problems. Traditional algorithms like Q-learning, Sarsa, and DQN are designed for discrete action spaces. DDPG is an extension of the DQN algorithm, enabling it to address the complexities of continuous action control [20].

The target motion data and the AUV state variables are used as inputs to the action network. The network then generates control variables for the AUVs to interact with the virtual environment. Considering the task objectives, a reward function is designed to guide the training process and motivate the AUV to complete the specified tasks in its surrounding environment. The network training pseudo-code is shown in Algorithm 2:
**Algorithm 2:** DDPG algorithm based on the rounding network training/*Initialization*/
(01)Initialize network parameters $\theta_{\mu }$ of the Critic network $Q(s,a|\theta_{Q})$ sand the Actor-network $\mu (s|\theta_{\mu })$ ;
(02)Copy the parameters of Critic and Actor to the corresponding target network:(03) 
$\theta_{Q^{\prime }}\leftarrow \theta_{Q}$ ⋯ $\theta_{\mu^{\prime }}\leftarrow \theta_{\mu }$ ;(04)Initialize the Reply Buffer R. /*Main Loop*/
(05)**For** episode = 1:M  /* M is the number of training times*/(06)  Initialize a random process N to add noise to the behavior;(07)  Initializes the state $s_{1}$ ;(08)  **For** t = 1:T  /* T is the max time step in each training process*/(09)    Get action based on the current strategy and explore the noise:(10)    
$a_{t}=\mu (s_{t}|\theta_{\mu })+N_{t}$ ;(11)    The output of the action network $a_{t}$ is transformed into the control variable of the AUV and integrated into the dynamic equation to obtain the reward $r_{t}$ and the next state $s_{t+1}$ ;(12)    Convert states to sequences $\left(s_{t},a_{t},r_{t},s_{t+1}\right)$ and store them in Reply Buffer R;(13)    Randomly sampled from the Reply Buffer as training data for the Actor-network and Critic network;(14)    
$y_{i}=r_{i}+Q^{\prime }(s_{i+1},\mu^{\prime }(s_{i+1}|\theta_{\mu^{\prime }})|\theta_{Q^{\prime }})$
(15)    Update Critic network parameters by minimizing the loss function L :(16)    
$L=\frac{1}{N}\sum_{i}\left(y_{i}-Q(s_{i+1}|\theta_{\mu^{\prime }})|\theta_{Q^{\prime }}\right){)}^{2}$
(17)    Calculate the sample policy gradient $\nabla_{\theta_{\mu^{\prime }}}\mu |s_{i}$ , and update the parameters of the Actor-network $\theta_{\mu }$ :(18)    
$\nabla_{\theta_{\mu }}\mu |s_{i}=\frac{1}{N}\sum_{i}\nabla_{a}Q(s,a|\theta_{Q}){|}_{s=s_{i},a=\mu (s_{i})}\nabla_{\theta_{\mu }}(s|\theta_{\mu }){|}_{s=s_{i}}$
(19)    Update the target network parameters $\theta_{Q^{\prime }}$ and $\theta_{\mu^{\prime }}$
(20)    
$\theta_{Q^{\prime }}\leftarrow \tau \theta_{Q}+(1-\tau )\theta_{Q^{\prime }}$
(21)    
$\theta_{\mu^{\prime }}\leftarrow \tau \theta_{\mu }+(1-\tau )\theta_{\mu^{\prime }}$
(22)  **End For**(23)**End For**

#### 2.6.2. Collaborative Rounding Environment

**Figure 11:**
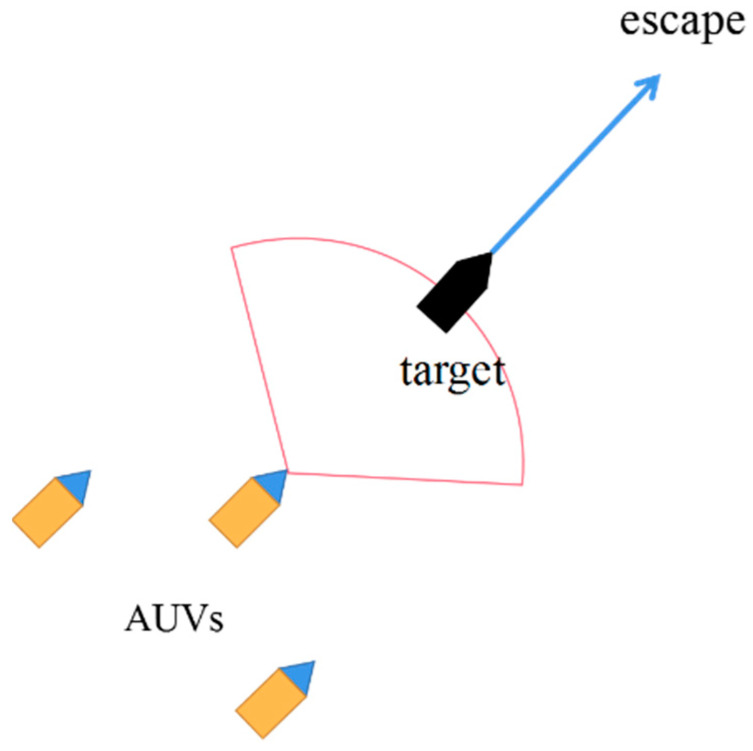
Schematic diagram of collaborative rounding.

Based on the DDPG approach, a dynamic equation-controlled virtual environment is constructed to train the agent network. This virtual environment facilitates the learning process. The schematic diagram of this setup is presented in Figure 11.

**Figure 12:**
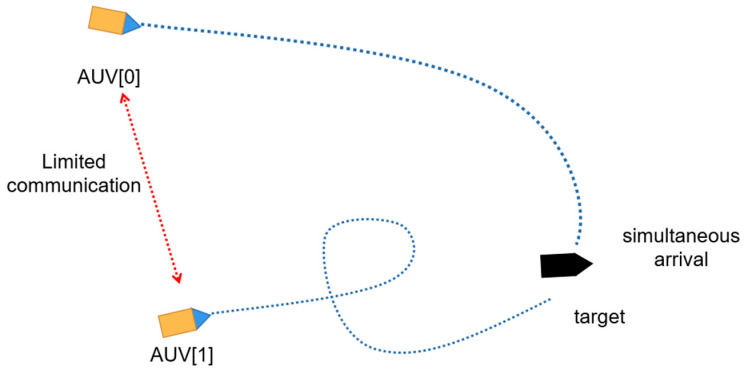
Simultaneous approach strategy in the limited communication environment.

Upon initializing the environment, a task target is assigned to the AUV according to its initial position. To optimize the likelihood of successful circumnavigation, the AUV should approach the target from various directions. Consequently, during the virtual environment’s initialization, each AUV is given a distinct orientation based on previous target position information. Figure 12 illustrates this process, serving as the foundation for establishing the reward function, this will be detailed in the subsequent sections.

During the entire training process, the position information and dynamic properties of AUV are used as state variables at each time step. The output layer of the Actor-network uses a sigmoid activation function. The resulting two output values are subtracted in order to obtain the normalized rudder angle output. This mechanism enables direct control of the AUV through the action network.

#### 2.6.3. Artificial Potential Field Reward

To enhance the success rate of circumnavigation during the training, a potential function is employed as a reward function. This function increases in value as the relative distance between entities decreases. 

For the *i*th AUV, the reward it faces can be expressed as:

(8) $$R_{i}=\left\{\begin{array}{l} +10\lambda ,\;if\;\Delta S_{i}<S_{\min}\\ +10\lambda e^{-\alpha \Delta S_{i}},\;if\;o_{i}\in T_{i}\\ -\lambda ,\;if\;w_{i}<\lim w \end{array}\right.$$

where $w_{i}$ indicates the angular velocity of rotation of AUV at the current time, $S_{i}$ indicates the distance between the AUV and the target, α is an adjustable distance factor, λ is the reward factor. $o_{i}$ indicates the location of the AUV in the environment, $T_{i}$ is an area controlled by two Angle functions: $T_{i}\in [\theta_{1}e^{\beta_{1}\Delta S_{i}},\theta_{2}e^{\beta_{2}\Delta S_{i}}]$ with two adjustable coefficients θ and β . 

**Figure 13:**
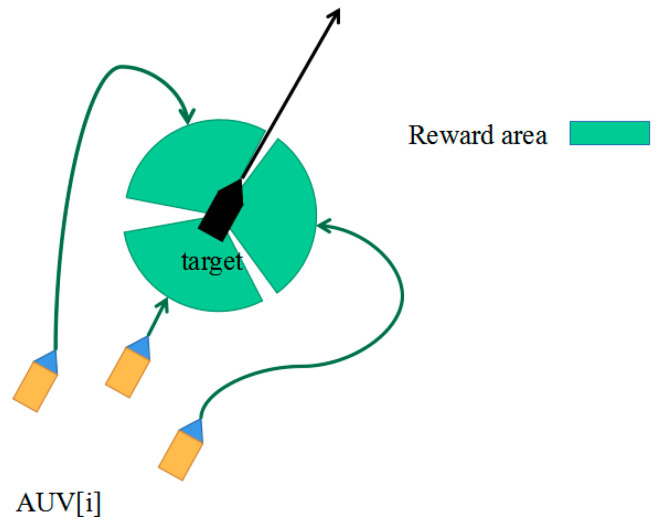
AUV formation surrounding attack reward map.

By using Formula (8), the reward *R* is obtained as the training data of the network. As depicted in Figure 13, by implementing the above three-tiered reward settings, the AUVs can be systematically guided to approach the target in the designated direction throughout the training process. The proposed approach also helps minimize sudden rudder angle fluctuations caused by the network output.

Compared to traditional simplistic approaches, our proposed method takes into account underwater communication and AUV dynamic constraints within the specified task scenario. Under the consideration of AUV communication and dynamic constraints, our method demonstrates superior performance in executing the overall task.

## 3. Simulation Results and Analysis

### 3.1. The Construction of Multi-Cooperative Task Virtual Environment

In this paper, the assembly of a complete AUV formation to perform a mission is termed a “mission”. Upon reaching the designated target area, multiple AUVs initiate long-range flights towards the predetermined detection area. This detection area is determined based on the platform analysis, and it is also the expected location. Considering the communication limitations and dynamic obstacles of the formation, the AUV formation executes this long-range flight while adhering to a predefined cooperative detection pattern. Upon detecting a target, its trajectory is predicted, facilitating the localization of the AUV formation. Following the establishment of optimal positions, the formation initiates tracking and pursuit once a target is determined. The pursuit commences from the position deemed most favorable. A 50 km × 50 km two-dimensional simulation environment and an AUV motion model are built in MATLAB, with the AUV performing duties at a set depth. The simulation running time is set to *T* = 10,000. The overall simulation procedure is divided into four phases: path planning, collaborative detection, target trajectory prediction, and surrounding and pursuit. Through these stages, the AUV’s behavior and interactions are systematically replicated in the defined environment.

### 3.2. Simulation Verification of the Proposed RRT Path Planning Algorithm

**Figure 14:**
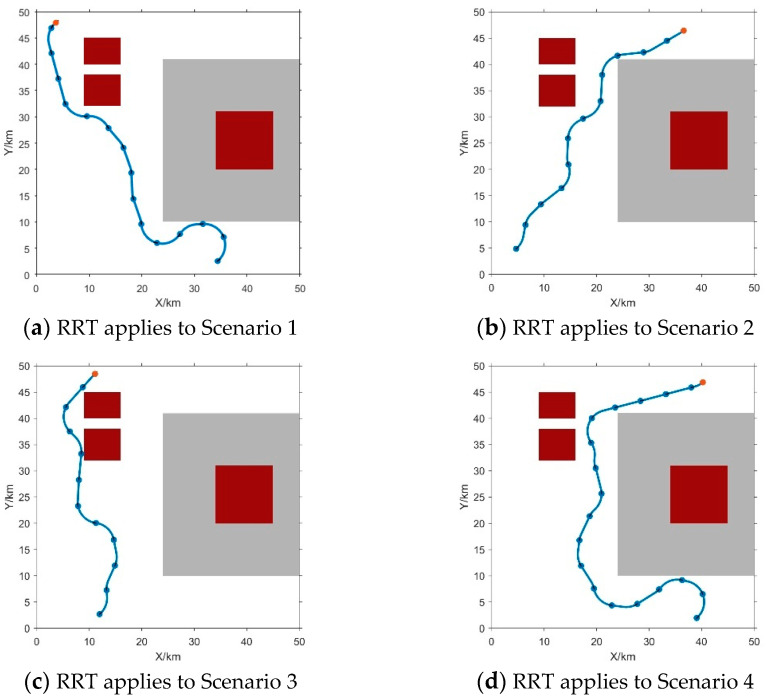
The RRT algorithm plots 4 paths in different situations. To assess the efficacy of the Rapidly Exploring Random Tree (RRT) algorithm, various task scenarios were devised for reliability verification. The red point signifies the target point, the blue point represents the AUV, and the blue curve outlines the planned trajectory. The gray area denotes the obstacle zone with a weight of 0.5, while the red area signifies the obstacle area with a weight of 0.9. In (**a**), the target occupies the upper left, with the Unmanned Underwater Vehicle (UUV) situated in the lower left. In (**b**), the target is positioned in the upper right, while the UUV remains in the lower left. (**c**) depicts the target in the upper left, and the UUV in the lower left. Finally, in (**d**), the target is located in the upper right, and the UUV is in the lower right. The consistent success across diverse scenarios underscores the robust applicability of the RRT algorithm.

Figure 14 shows the designed path of the AUV towards the virtual point, the proposed RRT algorithm can generate reasonable paths in basic obstacle environments, and its effectiveness can be verified. The positions of the target point are randomly assigned. The target point is marked by a red dot, and the AUVs are represented by blue dots. Obstacle areas are indicated by gray areas (with a weight of 0.5) and red areas (with a weight of 0.9 or higher).

The target point and AUVs are positioned diagonally, in order to evaluate the efficiency of the proposed RRT algorithm, and test the performance of the proposed algorithm to navigate these different starting points.

It can be seen from the blue trajectory in Figure 14, the proposed RRT algorithm has a powerful ability to accurately plan a seamless path for the AUV to reach the target point. Moreover, Figure 14 also shows that the path planned by the RRT expertly maneuvers around obstacles of different weights, resulting in a very smooth trajectory.

### 3.3. Formation Optimization

**Figure 15:**
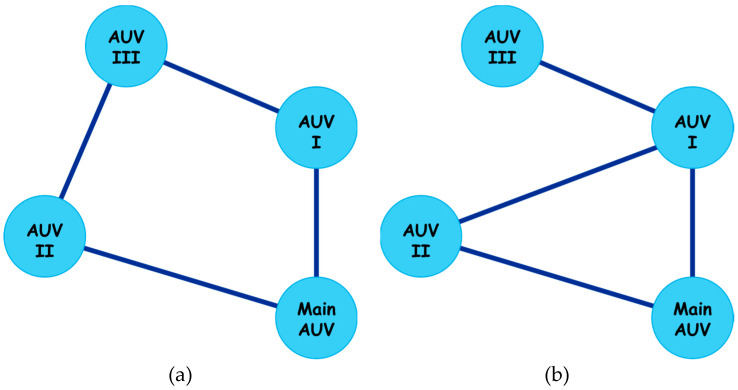
Two possible topological structures of the formation. (**a**) Each AUV establishes a communication relationship with its two connected AUVs, and the entire communication network is in a stable state; (**b**) AUV III only establishes a communication relationship with AUV I, so while AUV I, AUV II, and Main AUV can communicate stably, AUV III is unstable in the communication network.

To find the best formation for four AUVs, it is necessary to find the topological structure. Despite the additional communication relationships caused by the parameters, the preset topological relationships must be determined to achieve formation communication. There must be at least three edges in the formation communication network to connect four AUV nodes. However, considering the dynamic stability of the AUV and to expand the scope of design, one more edge should be added to improve the stability of the communication system. It is easy to see that every AUV can communicate with two other agents when the communication structure is a ring like in Figure 15a. But when it comes to Figure 15b, the AUV with only one communication relationship would be instability.

**Figure 16:**
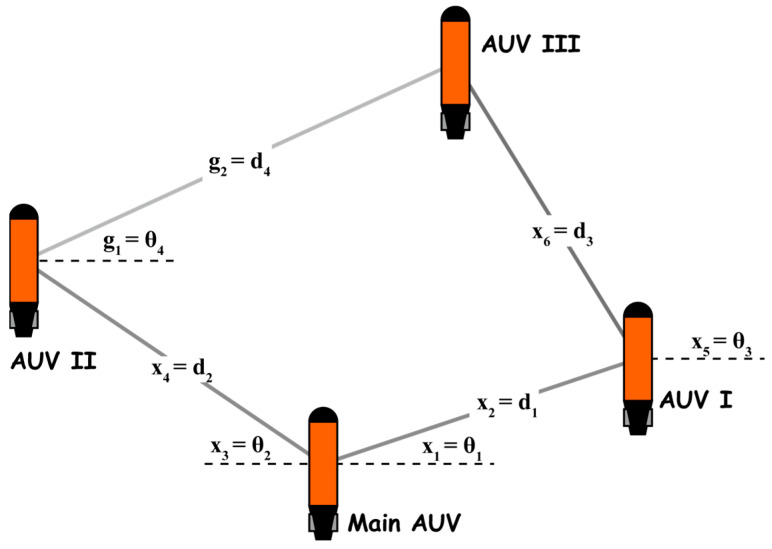
Parametric model of the formation structure.

When solving the formation optimization problem, it is necessary to parameterize the formation structure, so the polar coordinate method is considered. As shown in Figure 16, the polar coordinate system is established with the main AUV as the pole, and the coordinates of the two AUVs are determined by the pole angle and pole diameter. At the same time, the polar coordinate system is established with AUV I as the pole, and the coordinate of the AUV III is determined in the same way. At this time, the constraint of the optimization problem can be given according to the communication angle and distance of AUV II and AUV III.

In addition to the communication constraints determined by the topology, there are still some constraints to be considered in the communication links of the formation. First, to ensure the synchronization of communication, the communication of each node should be as consistent as possible. Secondly, when the communication signals received by a node are too close, it is easy to cause a signal interference. Therefore, it is also necessary to ensure that there is a certain angle between different communication signals.

There is always an inevitable error rate in AUV detection. When the target is covered in multiple AUV detection areas, the success rate can be effectively improved, expressed in the formula as follows.

(9) $$\Psi =1-\mu^{N}$$

In Formula (9), Ψ represents the success rate of formation detection, μ represents the error rate, and *N* represents the number of overlapping detection areas. Therefore, what needs to be considered in formation optimization is not only a larger detection area but also a more accurate detection effect. We define the feature detection area as follows and take it as the optimization goal:

(10) $$S^{*}=\sum_{i=1}^{N}S_{i}\Psi_{i}=\sum_{i=1}^{N}S_{i}\left(1-\mu^{i}\right)=S-\sum_{i=1}^{N}S_{i}\mu^{i}$$

**Figure 17:**
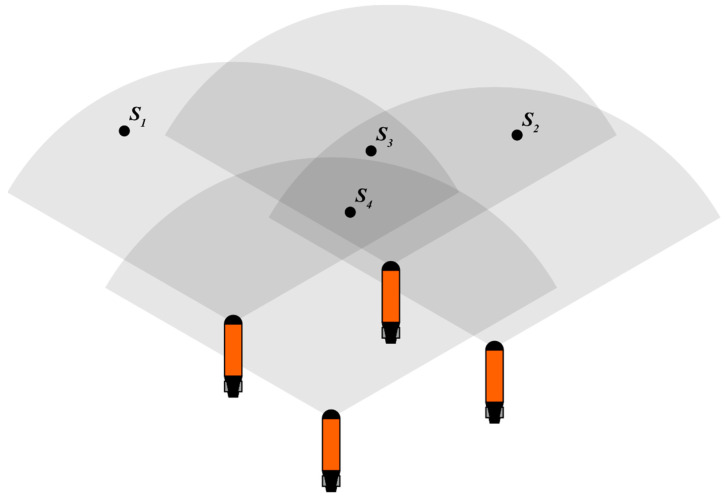
Schematic diagram of formation detection.

In Formula (10), $S_{i}$ means the area that can be detected by i AUVs, which can be seen in Figure 17. Combined with the communication link consistency agreement mentioned above, we can propose the target function of the optimization problem:

(11) $$f=\rho_{1}S^{*}+\rho_{2}R\left(\left\{d_{i}\right\}\right)=\rho_{1}S^{*}+\rho_{2}\left(\max\left(\left\{d_{i}\right\}\right)-\min\left(\left\{d_{i}\right\}\right)\right)$$

In Formula (11), $\rho_{1}$ means the coefficient of the characteristic area and $\rho_{2}$ means the coefficient of the extreme difference of the communication link. 

According to the above analysis, we put forward the format of the formation optimization problem and apply the particle swarm algorithm to solve the problem:

(12) $$\begin{array}{l} \min.\;f=\rho_{1}S^{*}+\rho_{2}\left(\max\left(\left\{d_{i}\right\}\right)-\min\left(\left\{d_{i}\right\}\right)\right)=f\left(\vec{x}\right)\\ s.t.\;{Ρ}_{0}= [\begin{array}{cc} 0 & 0 \end{array}]\\ \;\;\;{Ρ}_{1}= [\begin{array}{cc} x_{2}\cos x_{1} & x_{2}\sin x_{1} \end{array}]\\ \;\;\;{Ρ}_{2}= [\begin{array}{cc} x_{4}\cos x_{3} & x_{4}\sin x_{3} \end{array}]\\ \;\;\;{Ρ}_{3}={Ρ}_{1}+ [\begin{array}{cc} x_{6}\cos x_{5} & x_{6}\sin x_{5} \end{array}]\\ \;\;\;30{}^{\circ}\leq \langle {Ρ}_{i}-{Ρ}_{a},{Ρ}_{i}-{Ρ}_{b}\rangle \leq 150{}^{\circ},i\neq a\neq b\in \left\{0,1,2,3\right\}\\ \;\;\;40{}^{\circ}\leq \langle {Ρ}_{3}-{Ρ}_{2},\vec{oy}\rangle \leq 140{}^{\circ}\\ \;\left\{d_{i}\right\}=\left\{\left|{Ρ}_{1}-{Ρ}_{0}\right|,\left|{Ρ}_{2}-{Ρ}_{1}\right|,\left|{Ρ}_{3}-{Ρ}_{2}\right|,\left|{Ρ}_{0}-{Ρ}_{3}\right|\right\}\\ \;\;\;50\leq d_{i}\leq 2000\\ \;\;\;S^{*}=S-\sum_{i=1}^{N}S_{i}\mu^{i} \end{array}$$

In this problem, it is hard to calculate the overlapped area, so we used the Monte Carlo method for approximate calculation and selected the number of samples as 1000. To better balance the optimized goals of detection and communication, we defined the cooperation as follows:

(13) $$\left\{\begin{array}{l} \;\mu =0.8\\ \rho_{1}=-10^{-6}\\ \rho_{2}=10^{-3} \end{array}\right.$$

**Figure 18:**
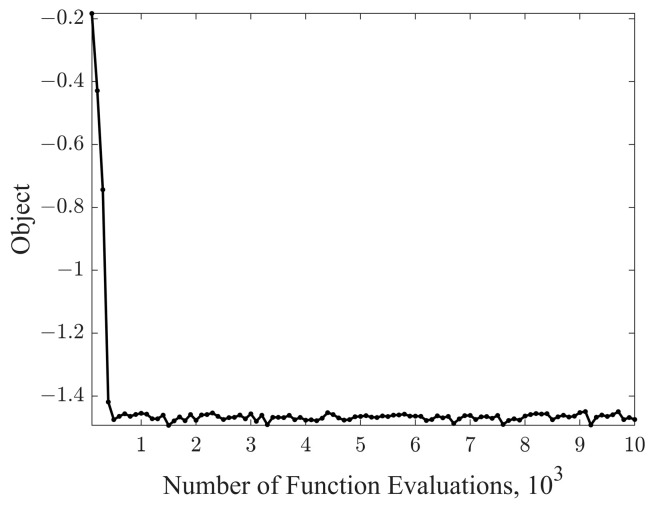
Iterative diagram of the target.

**Figure 19:**
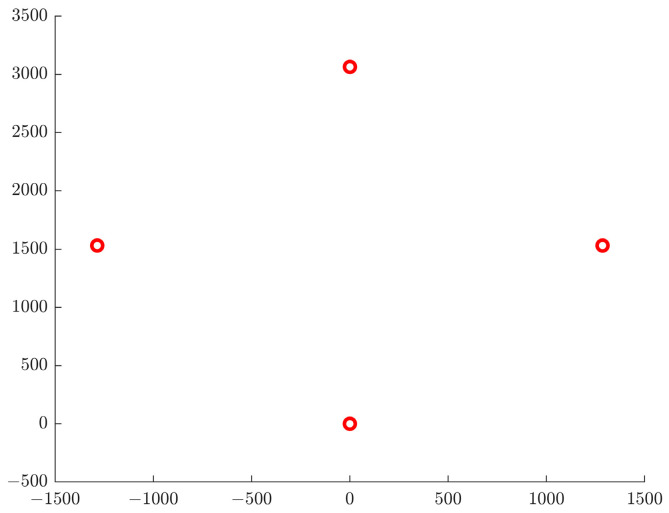
Optimized formation diagram. The four red circles represent the optimal locations for each of the four underwater vehicles.

To get a better optimization result, we tested the effect of different algorithms including GA and PSO with different inertia coefficients on this problem in Table 1. After 10,000 times of function evaluation of different algorithms, we got the following optimization results (Figure 18 and Figure 19): 

In Figure 19, the three red circles represent the optimized formation of the AUV fleet. Upon reaching the target area, the AUV fleet will reconfigure into the optimized formation, preparing for the next stage of collaborative search. 

### 3.4. Collaborative Detection and Dynamic Obstacle Avoidance

Considering the communication limitations of vehicles, a diamond formation with an internal angle of 60° is adopted in this paper. This formation mode will be applied when the AUV formation is established within the target water area with an initial span of 50 km. If there are hostile interference sources within the task area, the proposed model abstracts them into dynamic point avoidance. The dynamic boundaries are represented by black dots, and the fan-shaped regions depict the sonar detection areas.

When the sonar identifies a dynamic obstacle, the AUV formation immediately begins dynamic obstacle avoidance maneuvers. AUVs need to be able to avoid areas with dynamic obstacle distribution, in order to ensure that the AUV formation is always focused on target detection.

**Figure 20:**
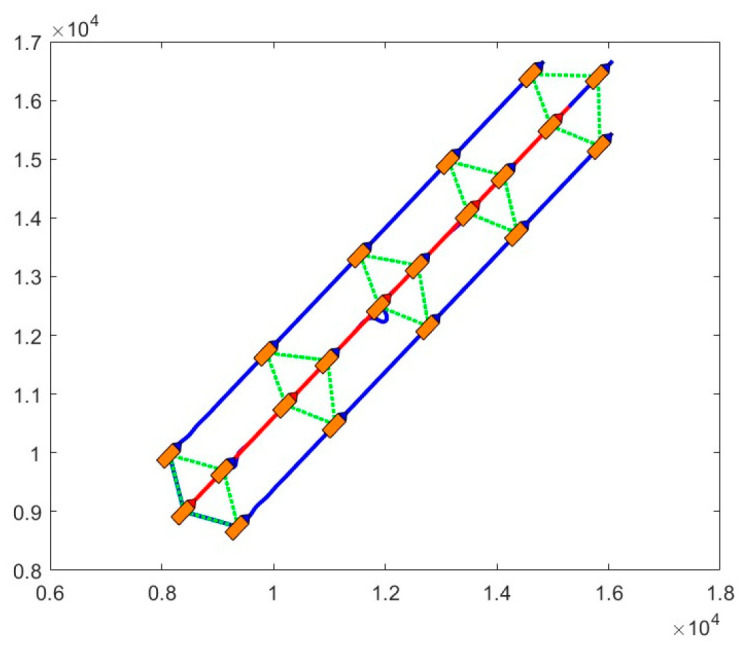
Navigation detection of AUV formations. This figure illustrates the AUV formation aligned with the optimized configuration for collaborative detection tasks. The dotted green lines symbolize the communication chain. The AUVs sporting blue heads acting as followers, while the blue lines depict the paths of the followers. The leader, distinguished by a red head, is denoted by solid red lines outlining its trajectory.

In Figure 20, four AUVs are configured in a diamond shape for target area detection. The red line represents the trajectory of the lead AUV, and the blue lines represent the trajectories of the three sub-AUVs. 

### 3.5. Trajectory Prediction with LSTM Network

In this section, the training method and the prediction effect of the LSTM network are illustrated in detail. In supervised learning methods, the selection of training data directly affects the performance of the trained network. Firstly, the target is independently run several times in the virtual environment to obtain enough target state data to construct the dataset. After that, the data in 11 consecutive time steps are taken as a set of inputs and output, where the first ten are set as inputs and the last one is set as output. The network uses the backpropagation method to update the parameters, and 20% of the dataset is taken as the test set to evaluate the loss function. In addition, during the construction of the training set, the maneuver angle of the target is set to 90 degrees, and 2% Gaussian noise is added.

To enhance the real-time performance of LSTM predictions, the rollout muti-step-ahead (RMS) [21] is applied to predict the target locations in the proposed method, which is expressed as Equation (14):

(14) $$[S_{target}^{t-W+1},\ldots ,S_{target}^{t}]\overset{M}{\to }{\hat{S}}_{target}^{t+1}\overset{M}{\to }{\hat{S}}_{target}^{t+2}\overset{M}{\to },\ldots ,\overset{M}{\to }{\hat{S}}_{target}^{t+U}$$

where $S_{target}^{t}$ demonstrates the target location information at the t step, W is set to 10 according to the training data, and M represents the generation method of the state information at the next time. When the target is in a detectable location, the state information at the next time step is filled with the true position of the target; otherwise, the state information is filled with the LSTM prediction position. 

**Figure 21:**
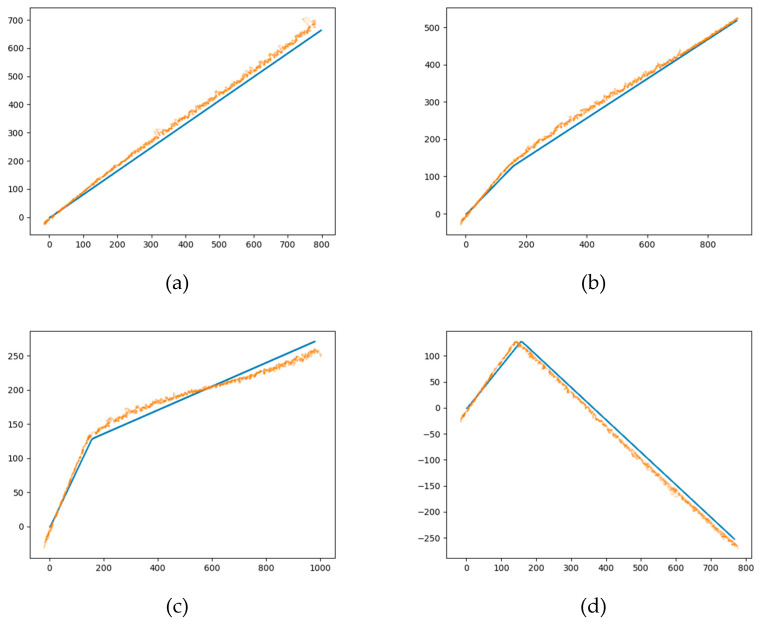
The prediction effectiveness obtained by the LSTM network on the conditions of different target maneuver angle. (**a**) The prediction effect of the LSTM network under the condition that the maneuver Angle of the target is 15 degrees, the orange dots and the blue dots correspond to the predicted and actual positions of the target respectively; (**b**) The prediction effect of the LSTM network under the condition that the maneuver Angle of the target is 30 degrees, the orange dots and the blue dots correspond to the predicted and actual positions of the target respectively; (**c**) The prediction effect of the LSTM network under the condition that the maneuver Angle of the target is 60 degrees, the orange dots and the blue dots correspond to the predicted and actual positions of the target respectively; (**d**) The prediction effect of the LSTM network under the condition that the maneuver Angle of the target is 90 degrees, the orange dots and the blue dots correspond to the predicted and actual positions of the target respectively.

Figure 21 illustrates the effectiveness of the LSTM network, which shows that the accuracy of the prediction position obtained by the LSTM network gradually increases with the target moves.

As shown in Figure 21, in the target maneuvering angles of 15, 30, and 60 degrees, the trajectory prediction obtained by the LSTM network is still effective. Different from the condition of 90 degrees which is used in the training set, these results demonstrate the generalization ability of the proposed method. In addition, the mean error of the prediction data is 19.4 m and the standard deviation is 7.2 compared with the real position under the 90-degrees maneuvering condition of the target.

### 3.6. Simulation Verification of the Target Surrounding Method

**Figure 22:**
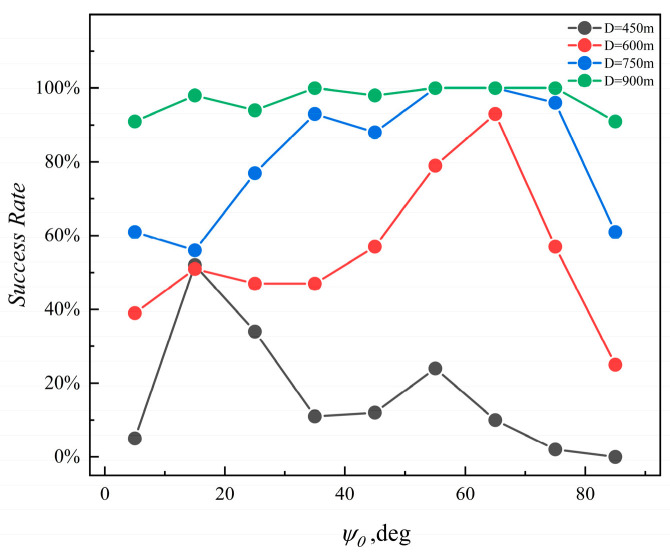
Multi-AUVs formation surrounding success rate.

Figure 22 shows a graph representing the success rate of target surroundings in various AUV formations. The abscissa represents the target orientation with the initial yaw angle of the AUV formation, and the ordinate represents the success rate of the AUV formation encircling the target. The four curves correspond to standard values for successful surrounding by AUV formations, which are set to 450 m, 600 m, 750 m, and 900 m, respectively. The key observation from Figure 22 is that, no matter where the target is in the AUV formation position, a high success rate of the surrounding can be achieved when the standard value is set to 900 m.

**Figure 23:**
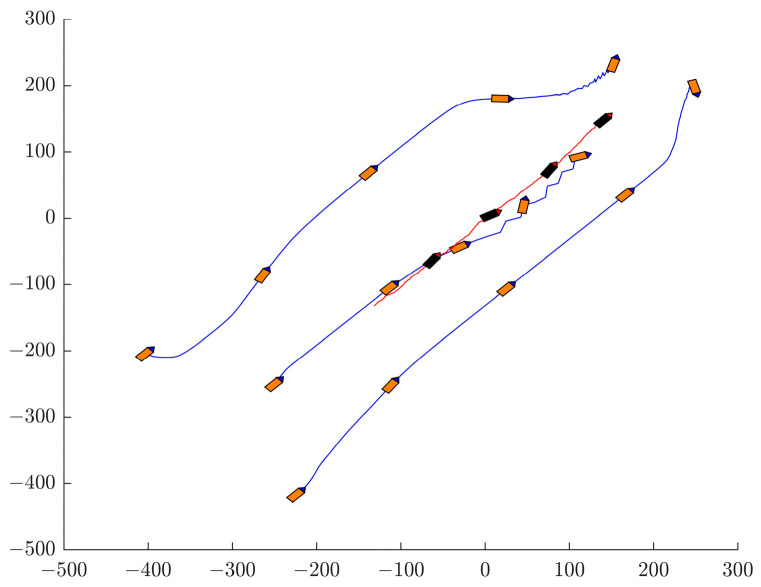
The process of AUV surrounding targets. This figure depicts the three orange AUVs completing a process for surrounding black target. The blue tracks outline their trajectories, while the red line shows the target’s motion.

As shown in Figure 23, the orange AUV represents the AUV formation, the blue trajectory represents the trajectory of the AUV formation, the black AUV represents the targets, and the red trajectory represents the target trajectory. Completion of the surrounding process involves the following steps. First, the AUV formation verifies the presence of the target. The AUV formation then adjusts to ensure it is in the best position before the target arrives. When AUV No. 1 identifies the target, it initiates communication with AUV No. 2 and AUV No. 3 on its flanks. In response, AUVs No. 2 and No. 3 are located in advantageous locations. At the same time, AUV No. 1 follows and pushes the target to the final stage of surrounding. The above surrounding process achieves successful completion of the target surrounding maneuver by the AUV formation. 

### 3.7. The Effectiveness Analysis of Surrounding Attack

**Figure 24:**
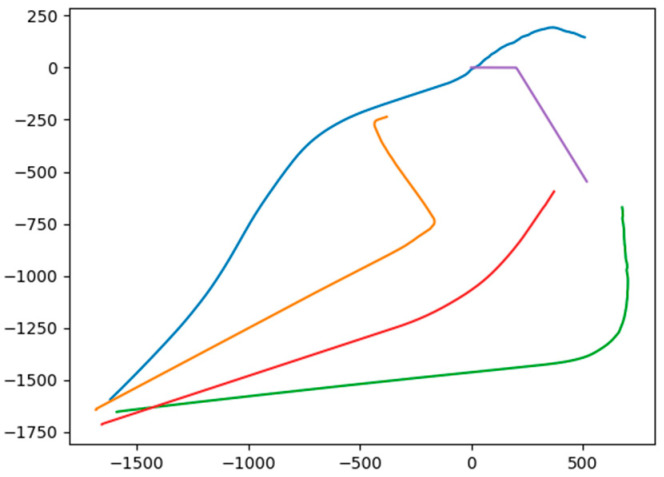
The AUV trajectory without using LSTM prediction on the condition of 8 knot and 60 degrees where the target is set as purple. The blue, orange, red, and green tracks depict the motion trajectories of the four AUVs.

**Figure 25:**
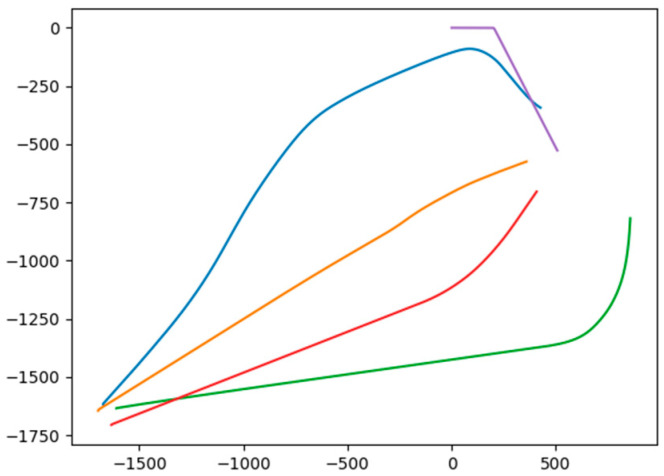
The AUV trajectory using LSTM prediction on the condition of 8 knot and 60 degrees where the target is set as purple. The blue, orange, red, and green tracks depict the motion trajectories of the four AUVs.

In this section, a test environment based on sparse probing is designed to verify the dependence of the proposed algorithm on LSTM in the sparse detectable environment. Basically, during each test, the AUVs have a certain 30% probability of losing the position state of the target at each time step. Based on this, we make a comparison between using the LSTM model to predict the target trajectory and not using the LSTM model. when the LSTM prediction is not used, the position state of the target under the detectable failure condition is set to zero as input to the actor network, and when the LSTM prediction is used, the input to the actor network is the prediction result of the LSTM network. In addition, the number of AUVs in our formation is set to four, and each set of data is set to run 100 times independently. The successful condition of the surrounding attack is set as when there are more than two AUVs within 200 m of the target. The success rate in different conditions is illustrated in Table 2, where nine instances of different conditions with three target velocities and three target maneuver angles are demonstrated. And the AUV’s trajectory on the instance of 8 knot 60 maneuver angle with not using the LSTM model and using the LSTM model is illustrated in Figure 24 and Figure 25, respectively.

According to the experimental results, the application of the prediction method based on the LSTM network improves the proposed surrounding attack method by a 10 to 20 percent success rate, which demonstrates the necessity of LSTM prediction in the process of surrounding. Furthermore, the AUV formation without LSTM prediction under the condition of sparse detection showed inferior performance, because as it gets closer to the target, the wrong position information makes some AUVs deviate from the target direction, which demonstrates the importance of LSTM prediction for formation synergy.

### 3.8. Discussion of AUVs Collaborative Environment

In the actual process of marine resources development, a safe and stable underwater environment is crucial. However, due to the underwater communication environment and the dynamic constraints of AUVs, the collaborative operation of AUVs is challenged greatly. In addition, a collaborative task often contains multiple mission objectives and requires planning different formation modes. Based on this, we propose a method of formation ferry, formation detection, and formation surrounding attack, which is suitable for the known partial target prior information, in order to cope with the complex task requirements of the formation control method, and based on this, a multi-cooperative task virtual environment can be simulated for multiple tasks. Nevertheless, the present study does not encompass the influence of ocean currents on AUVs and energy consumption within AUVs. In future endeavors, there is an opportunity to fortify our research in these particular domains.

## 4. Conclusions

This paper offers a comprehensive simulation of the entire mission execution process for a multi-AUV formation, including path planning, formation design methodology, collaborative search strategies, target trajectory prediction, cooperative surrounding techniques, and coordinated pursuit within multi-AUV formations. Firstly, this paper introduces a solution to the AUV path planning problem based on ocean information from the underwater platform. It develops a feasible path while considering the operational limitations of the AUV. Subsequently, considering communication limitations, this paper addresses the reconstruction and formation establishment of AUV formations. It utilizes an artificial potential field approach for formation navigation, ensuring robust and stable formation control. The article proposes a prediction scheme based on the LSTM neural network to predict the trajectory of the target after detection. This approach learns target motion characteristics, enabling the AUV formation to strategically surround the target during the decision-making process. After identifying the target location, the AUV formation initiates a surrounding mission. Finally, a high success rate in surrounding can be achieved when the standard value is set to 900 m.

In future work, we will consider more complex ocean environments to address the complexity of real-world ocean scenes and provide more practical guidance for AUV formations.

## Figures and Tables

**Table 1:** Optimization results.

Algorithm	Design Variables						Objective Variable
PSO (w = 0.4)	0.69	1358.97	2.37	1294.73	2.27	1529.89	−0.80
PSO (w = 1)	−0.87	2000.00	4.01	2000.00	4.01	2000.00	−1.46
PSO (w = 2)	−0.87	2000.00	4.01	2000.00	4.01	2000.00	−1.47
GA	−0.86	1865.29	4.01	1870.17	4.01	1860.75	−1.37

**Table 2:** Surrounding attack success rates using different trajectory prediction methods on 9 target instances. The black upward arrow signifies a rise in success rate, while the downward arrow denotes a decrease in success rate.

Target Velocity (knot)	Maneuver Angle	Not Using the LSTM Model Success Rate	Using the LSTM Model Success Rate
6	90	84% ↓	100% ↑
6	60	77% ↓	100% ↑
6	45	74% ↓	84% ↑
8	90	80% ↓	98% ↑
8	60	72% ↓	100% ↑
8	45	23% ↓	79% ↑
10	90	79% ↓	98% ↑
10	60	79% ↓	100% ↑
10	45	23% ↓	72% ↑

## Data Availability

Data available on request from the authors.
